# Nanotechnology in Dentin Disinfection: Can We Preserve the Bond?

**DOI:** 10.5005/jp-journals-10005-1559

**Published:** 2018

**Authors:** Prabhakar A Ramasetty, Amrita P Tripathi, Sugandhan S, Saraswathi V Naik, Deepak BM

**Affiliations:** 1-5 Department of Pedodontics and Preventive Dentistry, Bapuji Dental College and Hospital, Karnataka, India

**Keywords:** Ag-Au (silver-gold) nanoparticles, Chlorhexidine, Microleakage, Resin tag

## Abstract

**Aims:**

The aim of the present study is to evaluate the effect of cavity disinfection with 2% chlorhexidine (CHX) and Ag-Au nanoparticles on microleakage and resin tag penetrability of composite restoration under *in vitro* conditions.

**Materials and methods:**

Twenty-five human permanent molars extracted for therapeutic reasons were used in the study. Class V cavity of standard dimension was prepared on the buccal and lingual surfaces of the teeth. The teeth were randomly allocated into two groups based on the cavity disinfectant used; group I being 2% chlorhexidine gluconate (chlorhexidine FGM) and group II being cavity disinfectant containing Ag-Au nanoparticles (nanocare gold). In both the groups, the dentin was etched with 37% phosphoric acid and cavity disinfectants were applied following which dentine bonding agent and composite resin were applied and cured. The specimens were then viewed under stereomicroscope and scanning electron microscope for evaluation of microleakage and resin tag formation, respectively.

**Results:**

The results were statistically analyzed using independent ‘t’ test. No significant difference was seen between the two groups with respect to the microleakage and resin tag penetration values (*p* >0.05).

**Conclusion:**

Cavity disinfectant containing Ag-Au nanoparticles had no effect on the sealing ability and resin tag penetrability of composite resin in permanent molars when compared with 2% CHX.

**Clinical significance:**

Use of Cavity disinfectant containing Ag-Au nanoparticles with etch and rinse system can be preferred as it satisfies the ideal property of a cavity disinfectant which includes excellent antimicrobial action and non-detrimental effect on the sealing ability and resin tag penetrability.

**How to cite this article:**

Ramasetty PA, Tripathi AP, Sugandhan S, Naik SV, Deepak BM. Nanotechnology in Dentin Disinfection: Can We Preserve the Bond? Int J Clin Pediatr Dent, 2018;11(6):468-473

## INTRODUCTION

Worldwide, it is estimated that the replacement of an existing restoration accounts for 50–71% of each general dentist's activities.^1^ In most of the clinical situations, replacement of the restorations has been highly associated with the occurrence of secondary caries. It may be caused by residual bacteria left under the restoration, or by development of a microscopic pathway for leakage past the composite restoration due to polymerization shrinkage which may lead to degradation of bond, increased pulp sensitivity and pulpal inflammation.^2^ Hence, to reduce the development of secondary caries use of cavity disinfectant has gained a wider acceptance.

Among the various cavity disinfectants, chlorhexidine (CHX) has been widely used because of its antimicrobial property.^3^ It also has an inhibitory effect on the MMPs (against MMPs 2, 8, 9) in the dentin which can be useful in preventing collagen degradation and disintegration of the bonding interface over time.^4^ Contrary to this, few studies have shown that previous dentin treatment using chlorhexidine impaired the adhesion.^5,6^ In addition to the above mentioned adverse effect on adhesion, over the years, the antimicrobial property of CHX has been questioned through various studies.^7,8^

Recently, silver nanoparticles (NPs) due to its strong antibacterial activity are recommended for cavity disinfection before restoration. Nanoparticles of silver and gold have diversiform size and shape of different surface energies which ensures its antibacterial property against a different type of bacteria. Though these materials have good antimicrobial and antifungal activity, there are possibilities that the nanoparticles may agglomerate and create an adverse effect on bonding.^9^

To explore this possibility, the present study was conducted to compare and evaluate the effects of cavity disinfectant containing chlorhexidine (CHX) and Ag-Au nanoparticles on the sealing ability and resin tag formation of composite resins.

## MATERIALS AND METHODS

The study protocol was approved by the Ethics Committee at Institutional Review Board (under the number 434/2015–2016).

- *Study design:* Experimental, *in vitro*, between-group study
- *Sample size determination:* Based upon the information available from previous studies^6^ population means of the experimental and control groups were set at 110 and 90, respectively with the power of 0.8. The type I error probability was set at 0.05 to obtain a sample size of 15/group for microleakage. Additional 10 samples were collected for evaluation of resin tag penetrability.

### Selection of Teeth

Twenty-five human permanent molars extracted for therapeutic reasons were used in the study. The sample that was free of caries, white spots, cracks, and restoration were selected.

### Preparation of the Extracted Teeth

The teeth were cleared of debris and stored in a physiological saline solution containing 0.1% thymol at 37°C.

### Evaluation of Microleakage

Class V cavity of standard dimension was prepared on the buccal and lingual surfaces of 15 permanent molars tooth using a high-speed airotor handpiece with a straight fissure no. 9 bur and then, the teeth were hemisectioned mesiodistally with a diamond disc to obtain 30 specimens. The cavities were standardized with William's periodontal probe. Each cavity was approximately 2 × 1.5 × 6 mm, paralleling the cement-enamel junction. The preparations were extended 0.5 mm gingivally below the cementoenamel junction (CEJ). Each tooth sample was rinsed for 20 seconds with distilled water and dried with a blast of compressed air for 5 seconds. Caution was taken not to over dry the preparation.^10^ The samples were distributed into two experimental groups, consisting of 15 cavities each. All cavities were restored as given below:

In group I CHX (FGM, chlorhexidine), the cavities were etched with 37% phosphoric acid (Scotchbond) for 20 seconds, rinsed for 20 seconds with air-water syringe, and dried with compressed air for 10 seconds. This was followed by application of 2% chlorhexidine disinfectant on the cavity walls and floor using disposable micro applicator tips for 60 seconds.^11^ Then, bonding agent (prime and bond NT) was applied for 20 seconds, lightly air-dried for 5 seconds, and light cured for 10 seconds using LED light curing unit (BLUEDENT LED Smart) at pulsed mode. All the cavities were filled in 2 mm increments of composite restorative (FILTEK 350 XT, 3M ESPE). Each increment was cured for 20 seconds by a light cure unit. The composite restorations were finished in 24 hours after completion of restorations using diamond finishing bur (Diatech Coltene/Whaledent AG, Switzerland) underwater and were polished with Sof-lex Disks.

In group II, cavities were prepared in a similar manner as described previously. After preparation of the Class V cavity, etching was carried out with 37% phosphoric acid, as described in group I, Nanocare gold (Dental Nanotechnology, SA) was applied using disposable applicator tip in the amount of 5 drops and left undisturbed to evaporate for approximately 3 minutes. Following this, bonding agent (prime and bond NT) was applied and cavities were restored with composite restoration as described previously. All teeth were stored at 37°C for 24 hours in distilled water. The teeth were thermocycled in water between 5°C and 55°C with a dwell time of 30 seconds for a total of 500 cycles. After thermocycling, the root apices were sealed with modelling wax and two coats of nail polish were applied on the entire teeth specimen except 1 mm along the margin of restoration. The specimens were immersed in methylene blue dye at 37°C for 24 hours and later rinsed off thoroughly to remove residual dye. The teeth were sectioned buccolingually almost in the center of the restorations using a diamond disk attached to straight air motor handpiece. Microleakage was checked out using stereomicroscope (Leica Wild M3Z, Germany) at 30× magnification on both occlusal and gingival margins by a blinded examiner and the results were subjected to statistical analysis.

Meiers and Kresin, the scoring system, was used for evaluation of microleakage (1996):^3^

### Microleakage Scores

0 = no leakage
1 = penetration less than 0.5 the length of the occlusal/gingival wall.
2 = penetration greater than 0.5 the length of the occlusal/gingival wall.
3 = penetration up to and along the axial wall.

#### Evaluation of Resin Tag Formation

Ten extracted human permanent molars were selected. Class V was prepared on the buccal of all the teeth in a similar manner as performed for evaluation of microleakage. After cavity preparation, tooth samples were rinsed and dried for 20 seconds each. The cavities were etched with 37% phosphoric acid (Scotchbond) for 20 seconds rinsed for 20 seconds with air-water syringe and dried with compressed air for 10 seconds, this was followed by application of 2% CHX (Chlorhexidine, FGM) disinfectant on the cavity walls and floor in group I (n = 5) samples and nanocare gold in group II (n = 5) using disposable micro applicator tips. The method of usage of disinfectants was similar to that carried out for evaluation of microleakage. Following this, prime and bond NT (Dentsply) bonding agent were applied to the preparations according to the manufacturer's instruction and restored with composite resin (FILTEK Z 350 XT, 3M ESPE). The specimens were sectioned through the center of restoration (buccolingually) using a low-speed diamond and a continuous spray of water. Polishing of the specimens was carried out using silicon carbide paper of size 320 and 600 grit. Following polishing, the sections were immersed in 10% formalin for 12 hours and they were viewed under a scanning electron microscope (FEI Quanta 200), and measurement of resin tag length was evaluated in micron meters, and the results were subjected to statistical analysis.

## RESULTS

**Graph 1:**
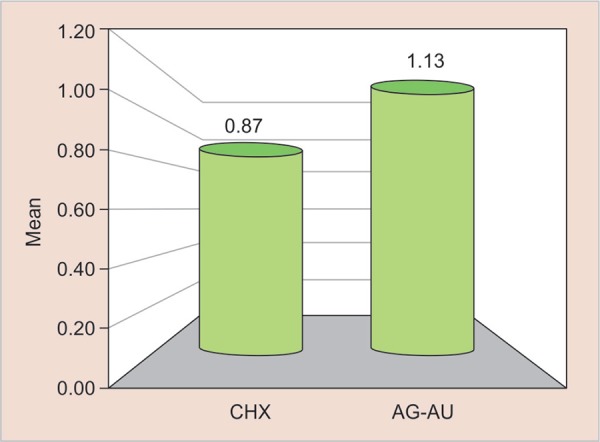
Mean comparison of microleakage between cavity disinfectant containing CHX and Ag-Au nanoparticles at the gingival margin

**Graph 2:**
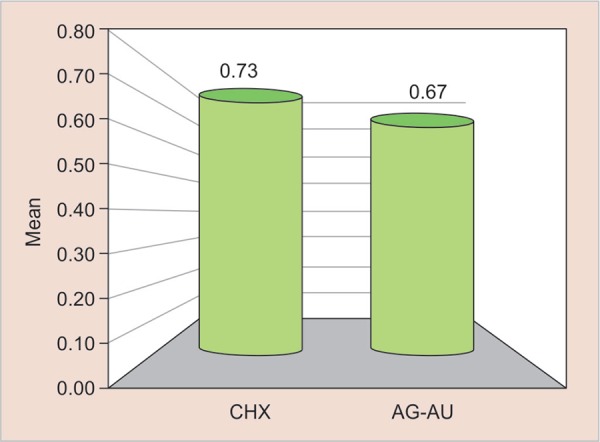
Mean comparison of microleakage between cavity disinfectant containing CHX and Ag-Au nanoparticles at the occlusal margin

All the analysis were carried out with SPSS 20.0 software. Means and standard deviations values of both the groups are shown in Tables 1 to 3. Independent ‘t’ test was used to compare the scores for microleakage on the occlusal and gingival margins. A *p* value of > 0.05 was established for all the tests. Graphs 1 and 2 shows the mean comparison of microleakage between CHX and Ag-Au nanoparticles at the gingival margin (*p* = 0.87 and 1.13) and occlusal margin (*p* = 0.73 and 0.67).

No statistically significant differences were found on pairwise comparisons for microleakage amongst the two experimental groups as shown in Graphs 1 and 2 with a *p* value of 0.363 and 0.702 at gingival and occlusal margins, respectively.

**Graph 3:**
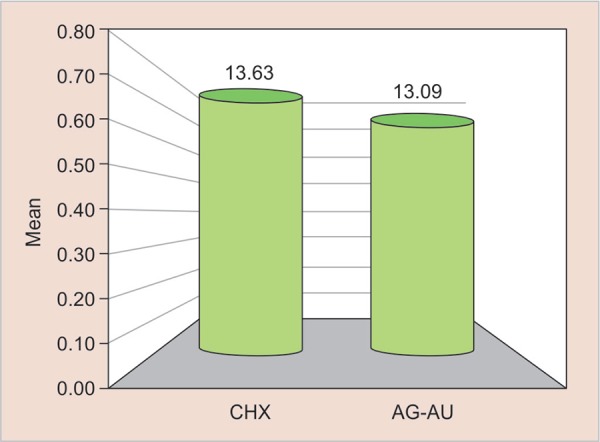
Mean comparison of resin tag formation between cavity disinfectant containing CHX and Ag-Au nanoparticles µm

For the resin tag formation, *p* value of 0.859 as shown in Table 3 was obtained which means that there was no statistically significant difference in the mean penetrability of both the groups. Table 3 and Graph 3 shows the resin tag lengths in both groups. Mean value of penetrability of 13.63 µm was seen in group I, whereas group II showed the mean value of penetrability of 13.09 µm.

## DISCUSSION

In the present study, following cavity disinfection with 2% chlorhexidine digluconate (chlorhexidine, FGM) and Ag-Au nanoparticles (nanocare gold), two important parameters, microleakage and resin tag penetrability were evaluated. Chlorhexidine which is a gold standard was selected as a control because it is one of the most commonly used antimicrobial agents in dentistry.

The results of the present study indicated that there was no statistically significant difference observed (*p* = 0.702, *p* = 0.363) with respect to the scores of microleakage between the two groups at the occlusal and gingival margin. When the enamel and gingival margin in the two experimental groups were compared, microleakage was higher in the gingival margin compared to the enamel margin. These results were consistent with the other two studies where there was more microleakage at the gingival margin. The rationale for this finding is due to thinner enamel margin at the gingival third than occlusal third and thickness of the incisal enamel prevented permeability resulting in a more resistant surface to dye penetration.^3,12,13^ With respect to the resin tag penetrability, it was found that there was no statistically significant difference (*p* = 0.859) between the lengths of the resin tags between the two groups. The length of resin tag formation in group I varied from 9.750 to 18.90 µm while in group II, it ranged from 7.01 to 15.89 µm (Micrographs 1 to 3).

**Table 1:** Mean comparison between cavity disinfection containing CHX and Ag-Au nanoparticles at gingival margin

*Variables*	*Groups*	*MIN*	*MAX*	*MEAN*	SD*	*Difference*	t **value	p*** value
Gingival	CHX	0.00	2.00	0.87	0.74	0.26 ± 0.09	0.925	0.363
	Ag-Au	0.00	3.00	1.13	0.83			NS

**Table 2:** Mean comparison between cavity disinfection containing CHX and Ag-Au nanoparticles at occlusal margin

*Variables*	*Groups*	*Min*	*Max*	*Mean*	SD*	*Difference*	t **value	p*** value
Occlusal	CHX	0.00	1.00	0.73	0.46	0.06 ± 0.03	0.386	0.702
	Ag-Au	0.00	1.00	0.67	0.49			NS

**Table 3:** Mean comparison of resin tag formation between cavity disinfectant containing CHX and Ag-Au nanoparticles in micron meters (µm)

*Variables*	*Groups*	*Min*	*Max*	*Mean*	SD*	*Difference*	t **value	p*** value
Resin tag formation (μm)	CHX	9.69	18.5	13.63	3.88	0.54 ±1.45	0.183	0.859
	Ag-Au	7.01	18.9	13.09	5.33			NS

*: Standard deviation;

**: independent ‘t’ test;

***: power of significance; CHX: Chlorhexidine; Ag-Au: silver and gold nanoparticles

**Micrograph 1:**
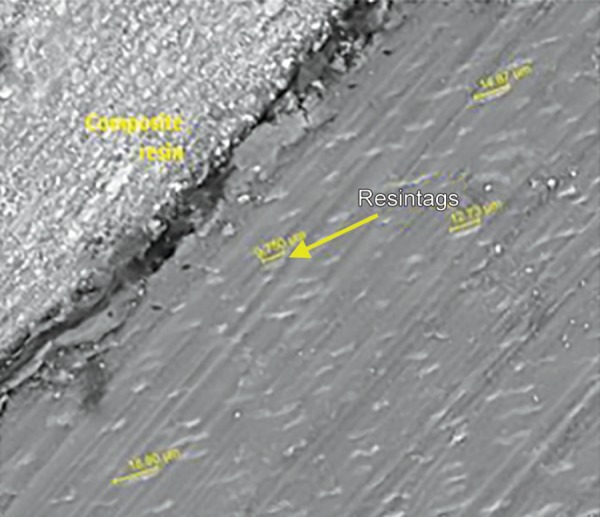
FEI scanning electron microscope evaluation showing length of the resin tag ranging from 9.750 µm to 18.90 µm (1000 × mag) in sample from group 1

**Micrograph 2:**
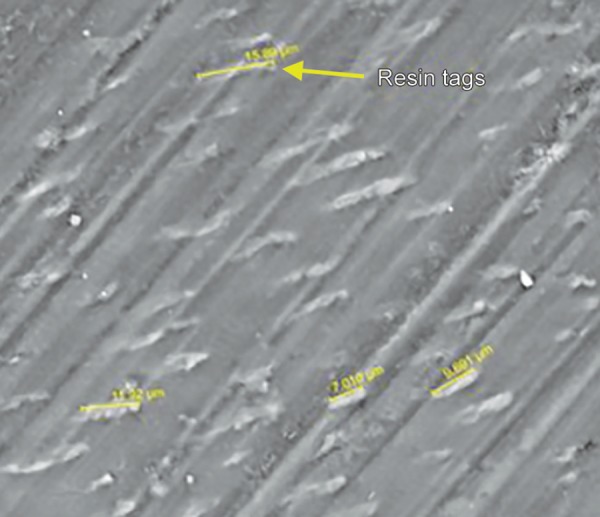
FEI Scanning electron microscope evaluation showing length of the resin tag ranging from 7.010 µm to 15.89 µm (2000× mag) in sample from group 1

**Micrograph 3:**
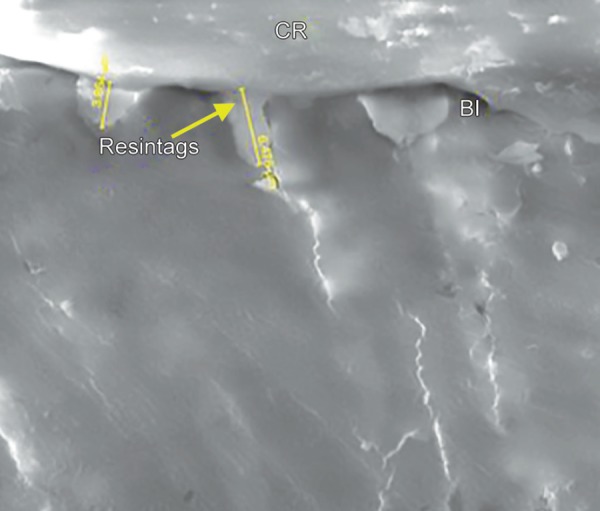
FEI Scanning electron microscope evaluation showing length of the resin tag ranging from 7.010 µm to 15.89 µm (2000 × mag) in sample from group 2

In the present study, nanocare gold cavity disinfectant did not affect the sealing ability and resin tag formation of the composite into dentin. The results of the study were in accordance with the study conducted by Porenczuk et al., where nanocare gold had no effect on the adhesive's bond strength.^14^ The reason for this could be that nanocare gold cavity disinfectant is composed of the numerous spherical nanoparticles (round, discoid) which were of the mean size of 48 nm. Lohbauer et al. suggested that the spherical shape of the nanoparticles provides with only one point of contact, so decreasing the tendency of agglomeration. In addition, the manufacturer claims that the metal nanoparticles are dispersed in a liquid carrier such as isopropanol.^15^ This provides an added advantage as NPs agglomeration can be prevented by their dissolution in a liquid carrier like methanol and isopropanol. Also, different sizes and shapes of nanoparticles may act as inorganic fillers similar to hybrid composites, and this may be the feature which enables nanocare gold to preserve the restorative material's physical properties.^9^

In the present study, the etch-rinse adhesive system was used. chlorhexidine and Ag-Au nanoparticles (nanocare gold) were applied after acid etching in solution form, since various authors have reported about benefits of good resin-dentin bond strength that were gained because of excellent property of chlorhexidine on acid-etched dentin-like strong positive ionic charge, ready binding to phosphate groups, strong affinity to the tooth surface and an increase in surface free energy of enamel.^16,17^

The bonding agent used in this study, prime and bond NT has acetone as a solvent that takes out the residual moisture, increases resin wetting, and prevents the adverse effects of organic contamination on bonding to tooth structure.

Microleakage was assessed because it is an important consideration in assessing the adhesion of materials to both enamel and dentin. As microleakage may be influenced by factors such as thermal changes; hence, in the present study, after composite restoration the specimens were subjected to thermocycling because it is a widely used method to simulate temperature changes that take place in the oral environment. In the present study, 500 thermal cycles between 50°C and 55°C were used. Radovic et al. also used 500 cycles, as it was based on the current ISO standard.^18^ Evaluation of resin tag was done in the present study because it shows the development of resin impregnated hybrid layer, which highly depends on the penetrating ability of resin into the etched dentin surface.

Reviewing the literature and correlating the inferences with the results of this study shows us that application of nanocare gold for cavity disinfection had no effect on sealing ability and resin tag penetration of the composite restorations. Further, *in vivo* studies needs to be conducted to examine the interaction and long term effect of nanocare gold with the other two step and self-etch adhesive system.

## CONCLUSION

Within the parameters tested in the present intervention, use of nanocare gold cavity disinfectants with etch and rinse system can be preferred because it did not have an adverse the sealing ability and resin tag penetration of resin restorations.
